# Magnetically Sculpted Microfluidics for Continuous-Flow Fractionation of Cell Populations by EpCAM Expression Level

**DOI:** 10.3390/mi17010009

**Published:** 2025-12-22

**Authors:** Zhenwei Liang, Xiaolei Guo, Xuanhe Zhang, Yiqing Chen, Chuan Du, Yuan Ma, Jiadao Wang

**Affiliations:** 1 Department of Mechanical Engineering, Tsinghua University, Beijing 100084, China; 2 Center for Medical Device Evaluation, National Medical Products Administration, Beijing 100081, China; 3 LenCyte Biotechnology (Xuzhou City) Co., Ltd., Xuzhou 221000, China

**Keywords:** magnetically sculpted microfluidics, microfluidic cell sorting, continuous-flow fractionation, EpCAM expression level

## Abstract

Continuous-flow separation of magnetically labeled cells according to surface-marker expression levels is increasingly needed to study phenotypic heterogeneity and support downstream assays. Here, we present a microfluidic platform that uses spatially engineered soft magnetic strips (SMS) to sculpt lateral magnetic deflection fields for quantitative, label-guided cell fractionation. Under a uniform bias field, the SMS generates controllable magnetic gradients within the microchannel, producing distinct lateral velocities among EpCAM-labeled tumor cells that carry different Dynabead loads, which indirectly report membrane protein expression. Multi-outlet collection converts these “race-based” trajectory differences into discrete expression-level-resolved fractions. A COMSOL–MATLAB framework and a force-equivalent metric |(***H***·∇)***H***| are used to optimize key structural parameters of the magnetic interface, including strip thickness, width, and vertical spacing from the flow channel. Three journey nodes at 1.5, 3, and 9 mm along the flow path define a three-stage cascade that partitions MDA-MB-231, Caco-2, and A549 cells into four EpCAM-related magnetic subgroups: high (H), medium (M), low (L), and near-negative (N). Experiments show that the sorted fractions follow the expected expression trends reported in the literature, while maintaining high cell recovery (>90%) and viability retention of 98.2 ± 1.3%, indicating compatibility with downstream whole-blood assays and culture. Rather than introducing a new biomarker, this work establishes a quantitative magnetic-field design strategy for continuous microfluidic sorting, in which the spatial configuration of soft magnetic elements is exploited to implement expression-level-dependent fractionation in next-generation magneto-fluidic separation systems.

## 1. Introduction

The accurate separation of phenotypically distinct cell populations is critical for unraveling biological heterogeneity, advancing clinical diagnostics, and improving the efficiency of gene-editing strategies [1]. In oncology, for example, the analysis of circulating tumor cell (CTC) subtypes has shed light on the mechanisms of metastasis, as different CTC subpopulations exhibit diverse invasive and metastatic potentials [2,3]. Being able to resolve these populations according to surface-marker expression levels provides crucial insight into tumor progression and therapeutic vulnerability, thus informing early detection, prognosis, and individualized treatment strategies [4,5,6,7]. Similarly, in CRISPR screening, where precise gene perturbations must be linked to phenotypic outcomes, phenotype-based enrichment is indispensable: separating cells according to marker expression enables direct connections between specific gene edits and functional readouts [8,9]. In drug screening, evaluating how distinct phenotypic subpopulations respond to targeted therapies likewise depends on reliable, expression-resolved separation [10,11].

Over the years, numerous methodologies have emerged for cell separation, each leveraging distinct physical principles [12,13,14]. Dielectrophoretic sorting exploits differences in cellular dielectric properties [15,16], while electrophoresis-based separation utilizes differences in electrophoretic mobility [17,18]. Density-gradient centrifugation and acoustic-field sorting capitalize on cellular density and volume variations, respectively [19,20]. Magnetic separation techniques, by contrast, employ magnetic fields in conjunction with magnetically labeled cells [21]. Although many of these methods are highly effective, they are often optimized for binary “present or absent” classification. When more nuanced, expression-level-resolved discrimination is required, magnetically labeled approaches are particularly attractive: antibody-functionalized magnetic beads bind to specific cell-surface markers and convert phenotypic differences into measurable magnetic signals, thereby enabling gentle, highly specific cell sorting with excellent biocompatibility [21,22,23,24,25,26].

Current magnetic-field-based separation strategies can be broadly divided into static batch systems and continuous-flow microfluidic platforms. Immunomagnetic approaches such as Magnetic-Activated Cell Sorting (MACS) based on EpCAM have proven effective for isolating CTCs, yet their batch-mode workflow fundamentally limits throughput and does not readily support continuous, expression-resolved fractionation [27]. Many continuous magnetic separation studies have focused on qualitative guidance of cell trajectories, without providing a quantitative description of lateral motion as a function of local magnetic field gradients. In such designs, the magnetic field is typically treated as a qualitatively tunable parameter, which complicates rational optimization and limits the ability to systematically engineer expression-level-dependent separation profiles [28].

Microfluidic systems have therefore emerged as a promising platform for continuous and precise magnetic cell sorting [16,18,29,30]. A variety of microfluidic devices have been reported that sort CTCs or other targets based on EpCAM or related markers, using magnetic nanoparticles in combination with fixed field gradients to achieve graded deflection or stepwise capture [31,32,33]. More advanced micromagnetic architectures have introduced patterned micro-magnets, self-assembled permanent micro-magnets, and gradient magnetic ratcheting schemes to realize quantitative magnetic separation and purification of cell subsets. Ratcheting cytometry and related digital magnetic sorting platforms, for example, use spatially periodic magnetic energy landscapes and time-varying actuation to translate differences in magnetic loading into discrete positional shifts, enabling high-resolution purification of T-cell subsets and other magnetically labeled targets. These systems demonstrate that expression-based magnetic sorting is feasible and powerful. However, their reliance on complex magnetic tracks, dynamic drive waveforms, or fixed permanent-magnet patterns can make the lateral force landscape difficult to redesign, and they do not always provide a simple, transferable metric that links micro-magnet geometry to the resulting magnetic-force profile along the flow path [27,34,35].

As a result, there remains an unmet need for a magnetic-field design framework that is both quantitative and structurally simple: one that can (i) map soft-magnetic interface geometry to a spatially resolved lateral force landscape under a uniform bias field, and (ii) implement continuous, multi-outlet fractionation according to surface-marker expression level in a single pass. Such a framework would complement existing ratcheting and digital sorting approaches by emphasizing spatial field sculpting rather than time-varying actuation, and would be particularly attractive for engineering multi-stage “race-based” sorting processes in microfluidic channels.

In this context, we develop a magnetically sculpted microfluidic platform that integrates soft magnetic strips into a continuous-flow device to realize expression-level-dependent fractionation of EpCAM-labeled tumor cells. By exploiting the field-shaping capability of soft magnetic elements under a uniform external field, we establish controllable lateral magnetic gradients within the microchannel and use a force-equivalent metric |(***H***·∇)***H***| to guide the optimization of strip thickness, width, and vertical spacing. This design enables differential lateral deflection of cells bearing distinct Dynabead loads and, through a multi-stage cascade of journey nodes and outlets, converts these trajectory differences into discrete expression-level-resolved fractions. The focus of this work is therefore not to redefine biological subtypes, but to provide a generalizable magnetic-field design strategy for continuous microfluidic fractionation that can be adapted to diverse markers and target cell populations.

## 2. Materials and Methods

### 2.1. Cell Culture

MDA-MB-231, A549, and Caco-2 cell lines were obtained from the National Infrastructure of Cell Line Resource (Beijing, China). RPMI-1640 medium containing 10% fetal bovine serum (FBS, Thermo Fisher Scientific, Waltham, MA, USA) and 1% penicillin-streptomycin (Thermo Fisher Scientific, Waltham, MA, USA) was used for maintaining MDA-MB-231 and A549 cells. For Caco-2 cells, DMEM high-glucose medium supplemented with 10% FBS and 1% penicillin-streptomycin was employed. Cell cultures were kept in a humidified atmosphere containing 5% CO_2_ at 37 °C with 95% humidity.

### 2.2. Magnetic Immune Labeling

MDA-MB-231, A549, and Caco-2 cells at logarithmic growth phase were harvested and labeled with Calcein-AM (MCE, MedChemExpress, Monmouth Junction, NJ, USA; Cat. No. HY-D0041) through 10 min incubation at 37 °C. Subsequently, cells underwent three washing steps using PBS. The washed cells were then adjusted to a final concentration of 2 × 10^5^ cells/mL in PBS, followed by the addition of 15 µL anti-EpCAM Dynabeads (Thermo Fisher Scientific, Waltham, MA, USA; Cat. No. 10618D). The cell-bead mixture was incubated for 30 min at room temperature under gentle agitation. For the whole-blood two-phase experiments (used for MCF-7 visualization), commercial mouse whole blood was stored at 4 °C and used as received (anticoagulated), without plasma removal, RBC lysis, filtration, or other preprocessing. MCF-7 cells were spiked at ~1 × 10^5^ cells per 1 mL whole blood, and anti-EpCAM Dynabeads were added at 15 μL bead suspension per 1 mL blood prior to incubation under the same conditions described above.

### 2.3. Cell Viability Assay

To quantify whether chip-based sorting affects cell viability, Calcein-AM/PI staining was performed at two time points: before sorting (baseline) and after sorting (outlet fractions). MDA-MB-231 cells were adjusted to 2 × 10^5^ cells/mL. For the pre-chip measurement, a small aliquot of the suspension was stained with Calcein-AM and propidium iodide (PI) for 10 min at 37 °C and imaged to obtain the baseline viability *V*_pre_. The remaining suspension was then incubated with 15 µL anti-EpCAM Dynabeads for 30 min at room temperature under gentle agitation and processed through the microfluidic chip.

After sorting, samples from the H/M/L/N outlets were collected and stained again under the same Calcein-AM/PI conditions to obtain the post-chip viabilities *V*_post,_*_i_* for each fraction. Viability was computed as the percentage of Calcein-positive/PI-negative cells among all counted cells. An overall post-sorting viability *V*_post_ was calculated as a yield-weighted average of *V*_post,_*_i_* using the outlet recovery fractions. We further define the viability retention caused by sorting as *V*_post_/*V*_pre_.

### 2.4. Fabrication of SMS on Glass Substrates

To fabricate the soft magnetic strip (SMS) with desired morphological features via precision etching, glass substrates were first coated with a 1J85 soft magnetic alloy strip using epoxy resin adhesive. 1J85 is a Ni–Fe–Mo soft-magnetic alloy with Ni content of about 80 wt% and Mo of about 5 wt%. It exhibits high initial relative permeability (μ_r_ on the order of 10^5^), a saturation flux density Bs of approximately 0.75 T, and a very low coercive field (H_c_ ≲ 0.5 A/m), making it well suited as a high-permeability field-shaping element in our SMS design and COMSOL (version 5.6) modeling. A uniform layer of S1813 photoresist was then applied through spin-coating to create the lithographic template. After conducting exposure and development procedures according to the specified patterns, selective etching of the exposed soft magnetic regions was performed using peroxide-enhanced hydrochloric acid solution applied via uniform spray deposition. The residual photoresist on the soft magnetic strips, featuring precisely delineated patterns, served as a protective mask to shield the 1J85 material in the designated areas from chemical attack during etching. This systematic photolithographic process enabled the successful production of patterned 1J85 soft magnetic arrays with high geometric precision on glass substrates, providing accurate spatial modulation of magnetic field distribution critical for the operational performance of the magnetically sculpted platform.

A thin layer of SU8 2025 photoresist was subsequently deposited onto the SMS-patterned substrate and pressed against a polytetrafluoroethylene (PTFE) block under a controlled pressure of 1.25–1.6 kPa. The photoresist was cured by UV irradiation for 30 min to form an encapsulation layer. The thickness of this encapsulation was characterized using white-light interferometry, which enabled precise determination of the vertical separation between the top surface of the SMS array and the encapsulation layer. This spacing was later defined as the “vertical distance (D)” between the SMS and the microchannel floor.

### 2.5. Fabrication of Embedded SMS in PDMS Microchannels

To embed SMS arrays within polydimethylsiloxane (PDMS) substrates, cured PDMS blocks were first coated with a thin layer of liquid PDMS serving as an adhesive medium. The 1J85 magnetic strips were gently placed and bonded to the PDMS surface, followed by slow curing at room temperature. Photolithographic patterning with S1813 photoresist and subsequent selective etching in peroxide-enhanced hydrochloric acid were performed, analogous to the procedure described in Section 2.4, to define the desired SMS geometries. To precisely control the separation distance between the SMS and the microfluidic channel, an additional liquid PDMS layer was applied as an encapsulating layer. The assembly was pressed against a PTFE block at a pressure of 0.8–1.1 kPa and thermally cured at 50 °C for 2 h. This process ensured permanent encapsulation of the SMS within PDMS. The vertical separation between the SMS surface and the top surface of the PDMS substrate was again verified by white-light interferometry, following the same definition of “vertical distance (D)”.

The microchannel structures were subsequently fabricated using standard soft lithography. Silicon wafers patterned with SU8 photoresist served as molds, onto which liquid PDMS was cast. The cured PDMS microchannels were then irreversibly bonded to the PDMS slabs containing the embedded SMS under compression (1.5–1.8 kPa) and thermal curing at 50 °C for 2 h. The resulting device consisted of microchannels with SMS arrays encapsulated obliquely above the flow path.

### 2.6. Chip Design and Integrated Assembly

The effectiveness of continuous magnetic cell sorting hinges on the integration of precisely engineered magnetic field environments with robust microfluidic architectures. At the microscale, the predominance of laminar flow enables stable fluidic interfaces, which are critical for accurate particle and cell separation [36]. However, such stability must be coupled to a well-controlled lateral force landscape to achieve selective and reproducible deflection of target cells. Traditional designs that rely on static permanent magnets often suffer from limited spatial control: non-uniform fields and poorly defined sample boundaries can lead to fluctuating sorting conditions and diminished reproducibility.

**Figure 1:**
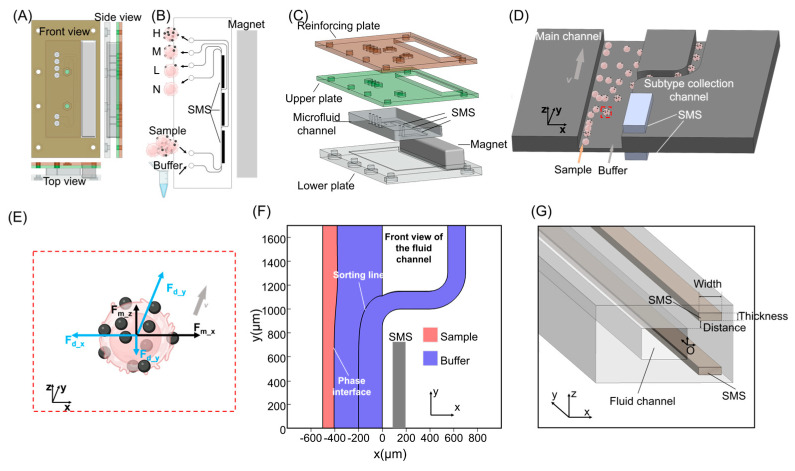
Design and operation of the microfluidic sorting chip: (**A**) Schematic of the chip structure. (**B**) Inlet and outlet configuration during operation. (**C**) Assembled chip with multilayer fixation. (**D**) Lateral deflection and outlet sorting under magnetic field. (**E**) Force analysis on cells experiencing magnetic and fluidic interactions. (**F**) Phase field distribution simulation results of sample and buffer streams within the microchannel, observed from front view. (**G**) Key structural parameters affecting magnetic field distribution in the vicinity of the microchannel: width (W) of SMS, thickness (T) of SMS, and vertical distance (D) from the microchannel to SMS. The origin point O for subsequent simulations and descriptions is positioned at the center of the channel on the side proximate to the SMS.

To address these challenges, we developed a composite microfluidic device that incorporates soft magnetic materials for magnetic-field reshaping and uses a precision-engineered multilayer acrylic compression frame to ensure robust assembly (Figure 1A–C). The PDMS microchannel layer was sandwiched between two precision-machined acrylic slabs and mechanically fastened to maintain alignment and provide leak-free operation. Dual SMS units were symmetrically embedded adjacent to the microchannel and magnetically biased by an external permanent magnet, which established an approximately uniform background field along the *x*-axis. Within this bias, each dual-SMS unit locally sculpted the magnetic field into a lateral gradient that induced expression-level-dependent deflection of EpCAM-labeled cells. Cells experiencing different Dynabead loads thus exhibited distinct lateral migration distances before reaching the outlet region. By cascading three such dual-SMS units in series, the device implemented a three-stage “race-based” fractionation process, in which the input sample was progressively partitioned into four outlets corresponding to distinct EpCAM-related magnetic subgroups.

### 2.7. Principle of Continuous Expression-Level–Dependent Cell Fractionation

The operating principle of the platform is to convert differences in cell-surface marker expression into differences in magnetic loading, and subsequently into distinct lateral trajectories under a sculpted magnetic field. In many immunomagnetic systems, membrane proteins serve as markers that distinguish phenotypically distinct cell populations. Under controlled incubation conditions, the number of antibody-functionalized Dynabeads bound per cell has been reported to scale with the abundance of the target antigen on the cell surface [24,37]. In this work, we leverage this relationship and treat the Dynabead load as an indirect, monotonic reporter of EpCAM expression level. Rather than redefining biological subtypes, our goal is to translate phenotypic differences in membrane protein abundance into well-controlled magnetic responses of cell–bead conjugates, and then to use these differences to realize expression-level-dependent fractionation.

To implement this concept in a continuous-flow format, we established a two-phase laminar flow within the microchannel, forming a stable interface between the sample and buffer streams. By setting the flow-rate ratio of sample to buffer at 1:4, the sample was confined to a narrow liquid column of approximately 100 μm width, thereby minimizing variability in initial lateral position. All cells were introduced within this sample phase, which served as a common “start line” for the race-like deflection process (Figure 1D). As cells convect downstream, the magnetically sculpted field generated by the SMS under a uniform bias induced lateral migration of Dynabead-labeled cells from the sample phase into the buffer phase. Under identical flow and field conditions, the lateral migration velocity was primarily governed by the magnetic force, and thus by the Dynabead load. Cells with higher EpCAM expression (and therefore larger bead loads) experienced stronger lateral forces and overtook lower-expression counterparts, causing their trajectories to diverge over the journey length. Multi-outlet collection at the channel terminus converted these trajectory differences into discrete expression-level-resolved fractions.

To capture the mechanics of cell motion within this environment, we performed a force analysis that accounts for both magnetic and hydrodynamic interactions (Figure 1E). A cell–bead conjugate primarily experiences three forces: fluidic drag, lateral magnetic attraction, and a vertical magnetic component that can pull cells toward the channel floor or ceiling. The lateral magnetic force is responsible for expression-level-dependent deflection. The vertical component, which becomes more pronounced near the SMS, can bring cells closer to the solid boundaries, where friction and possible intermittent contact reduce the effective lateral migration speed and, in extreme cases, may lead to transient stagnation. The magnitude of this vertical force depends on both bead load and cell height above the SMS. These effects were explicitly considered in the kinematic modeling to better reflect the diversity of cell behaviors under realistic operating conditions.

The hydrodynamic drag on a spherical cell of diameter *d*_c_ moving in a fluid of viscosity *η* is expressed as

(1) $$F_{drag}=3\pi \eta d_{c}(v_{f}-v_{c})$$

where ***v**_c_* is the cell velocity, and ***v**_f_* is the local fluid velocity. In the kinematic simulation, the tracked particle position corresponds to the centroid of each cell–bead conjugate, while the cell diameter *d_c_* affects migration through the drag term in Equation (1). In our microchannel, the flow operates in a low-Reynolds-number regime (Re ≪ 1), such that inertial effects and lift forces can be neglected and the velocity profile is well approximated as laminar and near-parabolic in the cross-section. We decompose motion into the axial (y) direction along the flow and the lateral (x) direction across the interface. While the lateral fluid velocity *v_f,x_* is negligible, the lateral drag ***F**_drag,x_* is approximately proportional to the lateral component of cell velocity *v_c,x_* relative to the local stream.

Upon magnetization in the applied bias field, each Dynabead behaves as an effective magnetic dipole whose magnetization ***M*** depends on the local magnetic field ***H***. In our modeling, this field dependence is explicitly taken into account by evaluating H from the COMSOL simulations and computing ***M***(***H***) according to the material’s magnetization response, rather than assuming a fixed, field-independent saturation magnetization. The magnetic force on a single bead in a non-uniform field can then be written in the standard form [38]:

(2) $$F_{m}=\mu_{0}(M\cdot \nabla )H$$

where *μ*_0_ is the permeability of free space, *χ* is the magnetic susceptibility, and ***H*** is the local magnetic field intensity. For a cell–bead conjugate carrying *N* beads, the net magnetic force scales approximately with *N* and can be expressed as

(3) $$F_{m}=N\mu_{0}V\frac{3\chi }{3+\chi }(H\cdot \nabla )H$$

where *V* is the total bead volume. Under a fixed bead material and operating range, the magnitude of this force is monotonically related to the scalar quantity |(***H***·∇)***H|***, which we therefore use as a convenient force-equivalent metric when optimizing the spatial magnetic-field landscape. Differences in bead load *N*, governed by EpCAM expression level, are thus mapped into differences in lateral magnetic force under a given field configuration. This relation underpins the race-based deflection mechanism used to achieve continuous expression-level-dependent cell fractionation.

For numerical implementation of the above force model, we constructed an explicit time-stepping kinematic simulation in MATLAB (version R2024) that is directly coupled to the COMSOL-derived flow and field distributions. Three-dimensional maps of the magnetic field and laminar velocity profile in the optimized device geometry were first computed in COMSOL and exported on a structured grid. In the simulation, each cell was represented as a spherical particle carrying a discrete number *N* of 2.8 μm diameter Dynabeads, consistent with the particles used in the experiments. The effective mass of the cell–bead conjugate and an effective hydrodynamic diameter were assigned based on the cell body and attached beads.

At the initial time, virtual cells were placed within the experimentally relevant sample band, uniformly distributed across the sample phase in the cross-section, while their initial velocity was aligned with the flow direction and set equal to the local fluid velocity. For each time step, the local magnetic field and flow velocity at the cell position were interpolated from the COMSOL data. The magnetic force, hydrodynamic drag and any additional wall-interaction contributions were then evaluated and combined to obtain the instantaneous net force on the cell–bead conjugate. Dividing this net force by the effective mass yielded the instantaneous acceleration, which was used to update the cell velocity and position via a forward Euler scheme. A time step of 1 × 10^−6^ s was used throughout, and at every step the forces were recalculated so that both the spatial variation in the magnetic field and the position-dependent flow profile were fully incorporated into the trajectory.

Cells were tracked until they either crossed the predefined sorting region associated with an outlet branch or exited the computational domain along the main channel. By repeating this procedure for different bead-loading levels, we obtained a family of simulated trajectories and corresponding journey distances. These time-resolved simulations provided the basis for selecting practical journey nodes along the channel, which were then used to determine the lengths and positions of the soft magnetic strip segments and the placement of the outlet channels in the experimental device.

### 2.8. Western Blot Analysis

MCF-7 cells were sorted into H/M/L/N fractions and pelleted by centrifugation. For the Western blot, a dilution-based sampling strategy was used to load an equal number of cells from each fraction (2.5 × 10^5^ cells per lane). Cell pellets were lysed in standard protein lysis buffer with protease inhibitors, and lysates were mixed with SDS loading buffer and denatured prior to electrophoresis. Proteins were separated by sodium dodecyl sulfate–polyacrylamide gel electrophoresis (SDS–PAGE) and transferred to a polyvinylidene difluoride (PVDF) membrane. After blocking, membranes were incubated with an anti-EpCAM primary antibody followed by a horseradish peroxidase (HRP)-conjugated secondary antibody, and bands were visualized by chemiluminescence.

## 3. Results and Discussion

### 3.1. Flow and Magnetic Field Distribution

Reliable expression-level-dependent fractionation in microfluidic channels requires a flow environment that is both spatially uniform and temporally stable. We therefore first validated the hydrodynamic conditions using COMSOL Multiphysics by solving the laminar Navier–Stokes equations in the full three-dimensional channel geometry. Under the flow rates used in this study, the Reynolds number based on the channel height and average velocity remained well below unity, indicating a strictly laminar regime in which inertial and lift forces were negligible and the velocity profile was near-parabolic across the cross-section. Within this regime, the interface between the sample and buffer streams remained sharply defined over the full length of the sorting region, providing a reproducible reference line for initiating magnetic deflection. Unless otherwise stated, all experiments were performed under this two-phase configuration with a sample–buffer flow-rate ratio of 1:4 and a total flow rate of 5 mL/h (sample phase: 1 mL/h; buffer phase: 4 mL/h), which defines the nominal operating condition used for device characterization and benchmarking. At the highest target-cell concentration used (2 × 10^5^ cells/mL), the sample-stream throughput corresponds to ~2 × 10^5^ cells/h (~56 cells/s) at 1 mL/h. The sample stream was confined within approximately 100 μm of the left wall of the microchannel (Figure 1F), and all cells entered the device within this narrow band, which served as a common “start line” for subsequent race-based deflection.

At each outlet, the flow field is adjusted such that a fraction of the buffer diverts into the branch channel while the remainder continues along the main channel. The separatrix between these two flow paths defines the “sorting line” for that outlet: only cells that have migrated laterally past this streamline are collected. In our design, the sorting line at the first outlet is positioned approximately 200 μm from the right boundary of the main channel (x = −200 μm in the coordinate system of Figure 1G). This combination of a well-confined sample band and geometrically defined sorting lines converts continuous lateral migration into a binary decision at each outlet and provides a clear geometric target for both simulation and experiment.

The magnetic field was then engineered to maximize lateral (x-direction) magnetic forces in the vicinity of the sample band while minimizing vertical (z-direction) components that could drive cells into the channel walls. This was achieved by tuning three structural parameters of the SMS: the strip width W, thickness T, and vertical distance D between the strip surface and the microchannel floor (Figure 1G). In COMSOL, the external permanent magnet was modeled as a uniform bias field oriented along the *x*-axis, such that the SMS primarily serves to reshape, rather than generate, the magnetic field within the channel.

Because the magnetic force experienced by beads within a magnetic field is influenced by both the intrinsic properties of the beads and the characteristics of the magnetic field itself [38], we introduced the scalar quantity |(***H***·∇)***H***| as a convenient force-equivalent metric for comparing different magnetic configurations independent of bead-specific properties. In our simulations, |(***H***·∇)***H***| is evaluated throughout the channel cross-section, and its lateral and vertical components are used to assess, respectively, the useful deflection force and the undesired tendency for wall attraction. Ideal field-shaping conditions correspond to large, spatially uniform lateral components with suppressed vertical maxima in the region occupied by the sample band.

**Figure 2:**
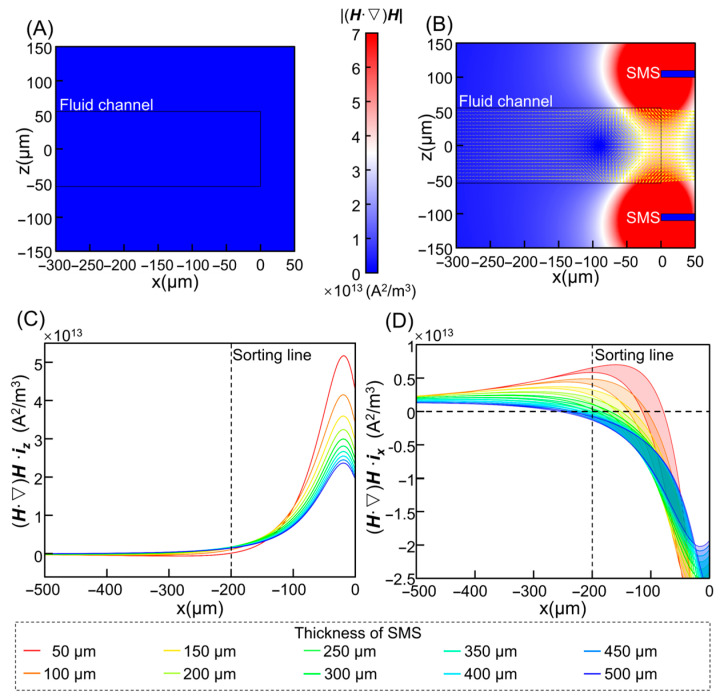
Effects of magnetic strip design on field shaping: (**A**) Magnetic force equivalent distribution |(***H***·∇)***H***| with only a background permanent magnet. (**B**) Enhanced field focusing and lateral force generation with dual soft magnetic strips adjacent to the microchannel. (**C**) Distribution of maximum vertical components of the (***H***·∇)***H*** parameter—proportional to magnetic force—along different x-coordinate positions within the microchannel. (**D**) Distribution of horizontal component ranges of the (***H***·∇)***H*** parameter in the magnetic field along different x-coordinate positions within the microchannel.

Simulations revealed that the external permanent magnet alone produces a relatively diffuse field with weak lateral gradients across the channel, insufficient to generate appreciable deflection within the available journey length (Figure 2A). Introducing symmetrically placed SMS on both sides of the channel dramatically reshapes the field, focusing magnetic flux into a narrow region near the sample band and generating strong lateral gradients while simultaneously reducing vertical components in the central portion of the channel (Figure 2B). The resulting |(***H***·∇)***H***| distribution shows a well-defined capture zone in which the lateral force increases smoothly as cells approach the SMS, indicating that the sculpted field can provide a predictable and spatially extended driving force for continuous deflection.

Figure 2C summarizes the maximum vertical components of (***H***·∇)***H***, and hence of the vertical magnetic force across the channel for different strip thicknesses. Large vertical peaks correlate with increased risk of cell-wall contact and frictional losses. Figure 2D shows the corresponding ranges of lateral components along the x-direction. Positive values indicate migration toward one SMS, negative values toward the opposite side, and zero crossings indicate positions where net lateral force vanishes. For each x-location, cells can occupy multiple heights z and thus experience a range of lateral forces; the colored bands in Figure 2D capture this distribution. For reliable sorting, the position at which the lateral component crosses zero must lie beyond the sorting line (x = −200 μm), ensuring that cells with sufficient magnetic loading can be driven past the collection boundary before the lateral force decays.

Overall, these simulations established that soft-magnetic field sculpting under a simple permanent-magnet bias can convert an otherwise weak and diffuse field into a spatially well-defined lateral force landscape tailored to the microchannel geometry. In particular, the SMS architecture offers designable, quantitative control over the lateral force profile, rather than merely boosting field strength as in a simple permanent-magnet configuration.

### 3.2. Optimization of Magnetic Parameters

**Figure 3:**
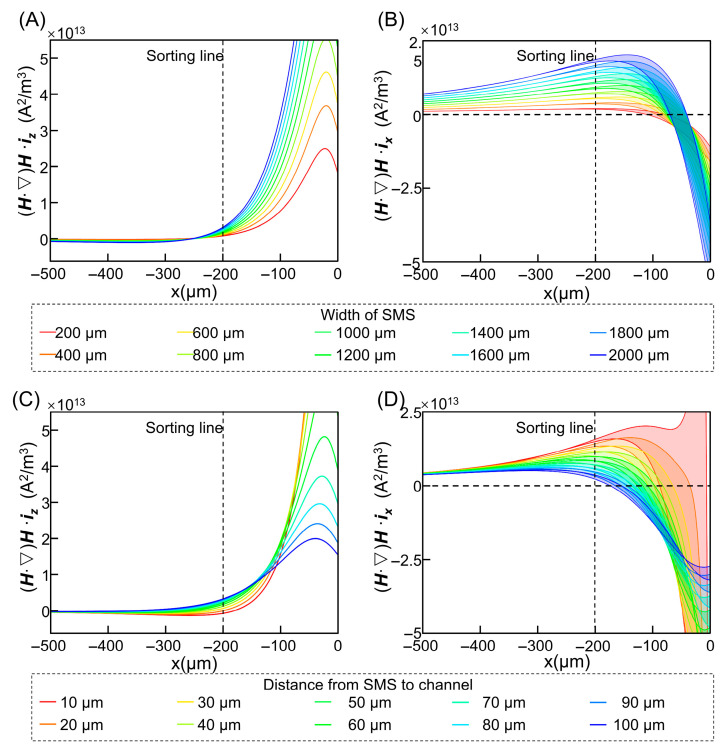
Influence of SMS parameters on force distribution: (**A**) Vertical components of (***H***·∇)***H*** vs. strip width. (**B**) Horizontal components of (***H***·∇)***H*** vs. strip width. (**C**) Vertical components of (***H***·∇)***H*** vs. vertical distance D. (**D**) Horizontal components of (***H***·∇)***H*** vs. vertical distance D.

To translate the above field-shaping strategy into a practical design, we carried out a parametric optimization of the SMS geometry, focusing on the width W and vertical distance D while fixing the thickness T at 100 μm. For each parameter combination, COMSOL simulations were used to compute the lateral and vertical components of (***H***·∇)***H*** across the sample region and to evaluate their spatial uniformity (Figure 3).

Increasing W enhances both lateral and vertical components of the magnetic force. While strong lateral forces are beneficial for rapid deflection, excessive vertical forces drive cells toward the walls, increasing friction and potentially leading to transient trapping or adhesion. Conversely, very narrow strips produce insufficient lateral forces, resulting in incomplete deflection within the available journey length. Similarly, decreasing D initially strengthens the field within the channel and improves confinement of the lateral force, but distances below ~50 μm introduce pronounced vertical inhomogeneities and sharp gradients, which can again promote wall interactions and non-uniform trajectories.

By balancing these trade-offs, we identified operating windows of W = 800–1200 μm and D = 50–70 μm that provide strong, smoothly varying lateral forces with limited vertical perturbations. Within this window, we further performed an orthogonal array optimization (Table S1) to identify the most robust combination. The configuration T = 100 μm, W = 1200 μm, and D = 70 μm yielded the most uniform lateral force field across the sample band, while keeping the vertical component below the threshold at which frictional effects become significant (Figure S1). This optimized design serves as the basis for all subsequent kinematic simulations and experiments and illustrates how |(***H***·∇)***H***| can act as an actionable design metric when tuning soft-magnetic interfaces.

### 3.3. Cell Motion Simulation and Sorting Results

**Figure 4:**
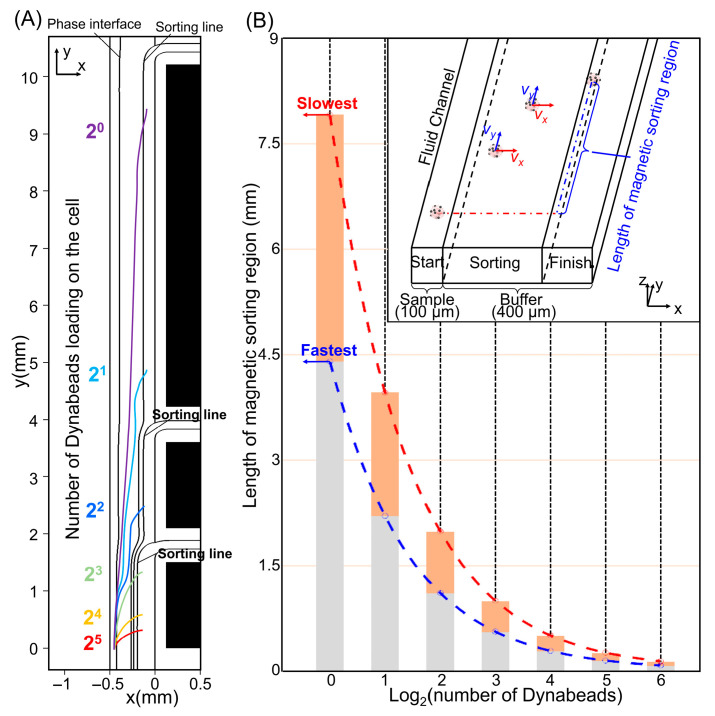
Kinematic analysis of cells with varying Dynabead loads in the sorting microchannel: (**A**) Centroid trajectories of cells bearing different quantities of Dynabeads. Cells exhibit differential responses to magnetic field influences, resulting in varying deflection efficiencies. (**B**) Magnetic field interaction distances required for cells with various Dynabead loads to achieve sorting criteria under fixed flow conditions. Due to boundary condition variations, cells with identical loading exhibit deviations in required interaction distances; therefore, ranges are represented as colored bands rather than discrete values.

Based on the force model and explicit time-stepping scheme described in the Methods, we first simulated the trajectories of EpCAM-labeled cells bearing different Dynabead loads as they traveled through the sculpted magnetic field. For each bead-loading level, virtual cells were initialized within the confined sample band, and their motion was propagated forward in time under the combined action of magnetic, hydrodynamic, and wall-interaction forces. Typical simulated trajectories show the expected race-based behavior: cells with higher Dynabead loads are deflected more rapidly away from the sample band, cross the phase boundary earlier, and reach the vicinity of the outlet branches sooner than weakly labeled or unlabeled cells (Figure 4A). Here, the plotted trajectories represent the centroid paths of the cell–bead conjugates; the cell diameter enters the dynamics through the drag term (Equation (1)), and the size sensitivity (15–20 μm) is summarized in Figure S2. In contrast, cells with low or negligible magnetic loading follow paths that remain close to the original streamlines and require much longer interaction lengths to approach the sorting region.

To capture the separation performance in a compact way, we introduced the concept of “journey distance”, defined as the axial distance a cell travels along the microchannel from its entry point until it first crosses the sorting region associated with an outlet branch, or until it exits the computational domain if it never crosses. For each discrete bead load *N*, the time-resolved simulations yield a distribution of journey distances that reflects variations in initial position and in near-wall interactions. Because Dynabead loading is monotonically related to EpCAM expression under matched labeling conditions, and expression differences are commonly represented on a log_2_ scale [39,40], we plotted the resulting journey distances as a function of log_2_(*N*) (Figure 4B). This representation clearly reveals four characteristic bands: cells with high bead loads cross the sorting region within short journey distances (high-expression group, H), moderate bead loads correspond to intermediate journey distances (medium group, M), weakly labeled cells require long interaction lengths before crossing (low group, L), and unlabeled or nearly unlabeled cells do not reach the sorting region within the available channel length and thus remain in the group N. To quantitatively relate the H/M/L/N labeling to bead loading under the fixed labeling protocol, we quantified the number of bound Dynabeads per cell in each outlet fraction and summarized the dominant bead-load intervals in Table S2 and the full outlet-resolved distributions in Figure S3. These measurements also quantify outlet purity, defined as the fraction of collected cells falling into the dominant bead-load interval assigned to each outlet.

Because wall friction and surface interactions can significantly influence lateral migration in high-gradient regions, we explicitly examined two limiting regimes: a minimum-friction case in which cells remain fully suspended in the bulk flow, and a maximum-friction case in which cells experience intermittent wall contact in the vicinity of the SMS. In both limits, the journey-distance bands corresponding to the H, M, L, and N groups remained well separated, although their absolute positions shifted slightly. This robustness indicates that the race-based deflection mechanism is not overly sensitive to moderate variations in near-wall behavior or surface properties, and that the group boundaries are primarily governed by magnetic loading rather than by subtle hydrodynamic differences.

On the basis of these simulations, we selected three representative journey nodes at 1.5 mm, 3 mm, and 9 mm along the flow direction. These nodes were chosen to lie between the journey-distance bands of adjacent expression-level groups, such that cells in the H group predominantly cross the sorting region before 1.5 mm, cells in the M group between 1.5 and 3 mm, and cells in the L group between 3 and 9 mm, while the N group remained largely unsorted within this length. We then mapped these nodes directly onto the device layout by segmenting the soft magnetic strip into three functional zones matching the chosen axial positions and by placing outlet branches at the corresponding locations. In this way, the time-resolved kinematic simulations provided a quantitative bridge between the lateral force landscape and the physical architecture of the device, enabling rational design of a three-stage cascaded sorter that implements expression-level–dependent fractionation in a single continuous pass.

To experimentally visualize the race-based deflection process under realistic labeling conditions, we first tested the magnetically sculpted sorter using MCF-7 breast cancer cells labeled with anti-EpCAM Dynabeads. In these experiments, whole blood was used as the sample phase and phosphate-buffered saline (PBS) as the buffer phase, so that both the hydrodynamic environment and optical contrast mimicked clinically relevant conditions. Under bright-field illumination, the interface between the whole-blood and buffer streams remained sharp and stable over the full length of the sorting region (Figure S4), in agreement with the laminar-flow simulations. Because the whole-blood and PBS phases remain sharply separated in the channel, endogenous blood cells (RBC/WBC) are expected to remain within the whole-blood sample phase and be transported predominantly to the downstream main-channel outlet (N), whereas the H/M/L branch outlets mainly receive buffer-phase fluid and magnetically deflected labeled cells. To verify outlet purity, we examined potential blood-cell carryover into the H/M/L fractions using CD45 staining for WBC and Ter119 immunostaining for RBC identification (with morphological confirmation under bright-field). No RBC/WBC events were detected in H/M/L across three replicate measurements (Table S3). Consistent with the bright-field observation of a confined whole-blood stream, the dark blood phase was visually observed to exit predominantly through the main-channel outlet (N), while the branch outlets appeared optically clear under bright-field imaging.

**Figure 5:**
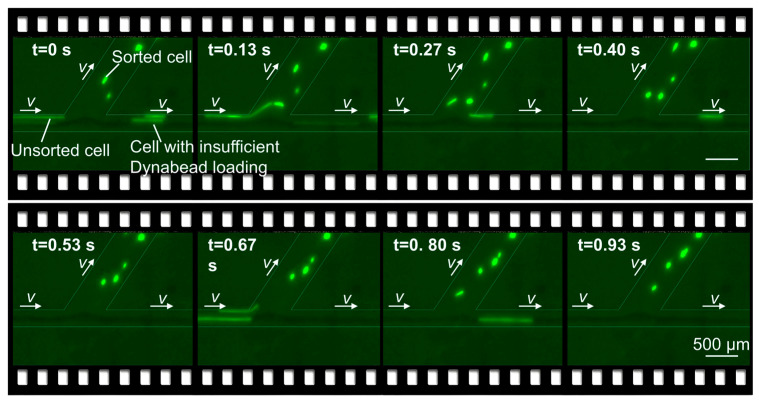
Fluorescence images of MCF-7 cells undergoing magnetic deflection and outlet collection in the continuous-flow chip. The main channel runs from left to right with lateral branch channels positioned centrally. During the sorting process, cells meeting the Dynabead loading threshold are directed into the branch channels, while cells with insufficient magnetic loading continue along the main channel toward subsequent magnetic control positions.

Fluorescence microscopy images acquired near the magnetic control region and outlet branches reveal the expected race-based behavior (Figure 5, Supplementary Video S1). Magnetically labeled MCF-7 cells bearing sufficient Dynabead loads undergo clear lateral deflection across the phase boundary and enter the branch channels, whereas weakly labeled or nearly unlabeled cells remain confined to the main channel and are transported downstream to subsequent control positions. Within the whole-blood matrix, numerous labeled cells successfully traverse the sample–buffer interface and are captured at their respective outlets, demonstrating that the sculpted lateral magnetic field can drive expression-dependent deflection even in viscous, optically dense media. These observations provide qualitative experimental confirmation of the kinematic model and show that the soft-magnetic field-sculpting strategy is compatible with continuous operation in clinically relevant sample environments.

Because lateral magnetic deflection is time-of-flight limited, we further evaluated the robustness of the kinematic predictions to variations in the main (total) flow rate by performing a quantitative flow-rate sweep while keeping the sheath-to-sample ratio fixed at 4:1 (Figure S5). The results show that increasing the total flow rate shifts the bead-loading thresholds upward and increases the required interaction length; for example, when the total flow rate increases from 5 mL/h to 8 mL/h, weak-label boundary leakage begins to appear and the H-capture threshold shifts from N ≈ 5 to N ≈ 7. Conversely, decreasing the total flow rate to 2 mL/h shifts thresholds downward and leads to over-capture, where most bead-labeled cells are predicted to be collected in the H fraction, and the L fraction becomes nearly empty (Figure S5). These trends support that the device should be operated around the nominal condition reported above, while Figure S5 defines the practical operating window and the expected failure modes when deviating from it.

In addition, we evaluated the influence of sheath confinement, because the sheath-to-sample ratio sets the start-line width and therefore the spread of initial lateral positions that seed the journey-distance bands. Using the same kinematic framework, we performed a systematic sweep of the start-line width to quantify how initial-position dispersion propagates into the journey-distance bands. The results (Figure S2B) indicate that narrower start-line confinement reduces the uncertainty envelope for a given bead load, but also proportionally reduces sample throughput at fixed total flow. Based on this trade-off, we used a ~100 μm start line (the nominal configuration) for experimental benchmarking.

### 3.4. Benchmarking EpCAM Expression-Level Fractionation Across Multiple Cell Lines

To evaluate the practical performance of the magnetically sculpted sorter under realistic labeling conditions, we benchmarked expression-level–dependent fractionation using three epithelial cancer cell lines: Caco-2, MDA-MB-231, and A549-that are known to exhibit distinct EpCAM expression profiles. In this study, the three lines were processed separately, not as a mixed sample, so that the device could be tested as an engineering platform for expression-resolved fractionation rather than as a tool for redefining biological subtypes across cell types. Each line was labeled with anti-EpCAM Dynabeads under the same staining protocol, introduced into the device, and passed through the three sequential sorting zones defined by the journey-distance nodes at 1.5, 3, and 9 mm.

**Figure 6:**
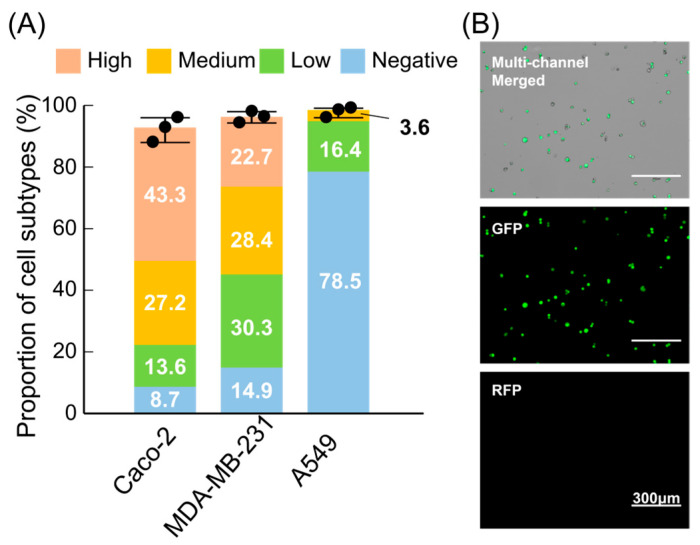
Benchmarking EpCAM expression-level fractionation across three epithelial cancer cell lines: (**A**) Outlet-resolved distributions of Caco-2, MDA-MB-231, and A549 cells (n = 3 per line). Bars indicate the relative proportions of high (H), medium (M), low (L), and near-negative (N) expression-level groups, as defined by the dominant outlet for each fraction. Distinct patterns across the three lines reflect their known EpCAM expression differences and reveal intra-line heterogeneity, including an EpCAM-low/negative subset within Caco-2. (**B**) Representative post-sorting viability assessment (MDA-MB-231) based on Calcein-AM/PI staining. The yield-weighted post-sorting viability was 98.0 ± 1.4% (n = 3); the corresponding viability retention *V*_post_/*V*_pre_ was 98.2 ± 1.3% (n = 3).

Statistical analysis of the outlet distributions (n = 3 per line) showed clear stratification into four EpCAM-related expression-level groups, which we denote as H (high), M (medium), L (low), and N (near-negative) according to the dominant outlet for each fraction (Figure 6A). Consistent with prior reports, Caco-2 cells were mainly recovered in the H and M outlets, MDA-MB-231 cells exhibited an intermediate distribution with appreciable L-fraction content, and A549 cells were enriched in the L and N outlets [41,42,43]. Importantly, a non-negligible fraction of Caco-2 cells appeared in the N outlet, indicating the presence of an EpCAM-low/negative subpopulation within this nominally high-EpCAM line. This illustrates that the race-based deflection mechanism is sensitive enough to resolve intra-line heterogeneity in surface-marker expression, not just coarse differences between cell lines.

Beyond marker-expression differences, cell-type-dependent physical factors can also modulate sorting outcomes. In our kinematic model, cell diameter enters the Stokes drag term (Equation (1)), so larger cells migrate laterally more slowly at the same magnetic loading; accordingly, the size-sensitivity analysis (Figure S2A) predicts only a modest shift in the H/M/L bead-load thresholds while preserving the monotonic ordering with bead load. Prior reports indicate that MDA-MB-231/MCF-7/A549 cells are typically ~15 ± 3 μm [44,45,46], whereas Caco-2 cells tend to be larger (~20 μm) [47,48,49], which may partially explain small shifts in outlet occupancy under a single device design and operating condition.

To directly confirm that the outlet-defined fractions indeed reflect graded EpCAM expression, we further performed Western blotting on MCF-7 cells collected from the H, M, L, and N outlets under the same labeling and sorting conditions (Figure S6). In the large-scale run used for this validation (2.0 × 10^6^ total MCF-7 cells), the collected yields were H ≈ 7.5 × 10^5^, M ≈ 4.0 × 10^5^, L ≈ 6.0 × 10^5^, and N ≈ 2.5 × 10^5^ cells. Importantly, for Western blotting, we loaded an equal number of cells from each fraction (2.5 × 10^5^ cells per lane, obtained by dilution-based sampling), so the monotonic decrease in EpCAM band intensity from H to N reflects a per-cell EpCAM signal trend rather than unequal cell numbers. Although we did not perform an orthogonal single-cell EpCAM quantification in this study, the outlet-resolved single-cell bead-count statistics (Table S2 and Figure S3) provide a direct validation of the labeling variable that governs magnetic deflection under the fixed protocol.

From an engineering perspective, two performance indicators are particularly relevant: overall recovery and expression-resolution. Across the three cell lines, the overall recovery-defined as the fraction of input cells collected in all four outlets-consistently exceeded 90% (n = 9), indicating that cell loss due to adhesion, trapping, or channel clogging is limited under the chosen operating conditions. The outlet-specific recovery fractions (H/M/L/N), defined relative to the input cell number, are reported in Table S4. At the same time, the distinct outlet distributions obtained for the three lines, together with the multi-outlet partitioning within each line, demonstrate that relatively modest differences in EpCAM expression and bead loading can be converted into robust differences in outlet occupancy. In other words, the platform functions as a continuous-flow “expression-ranking” device: higher EpCAM/Dynabead loads tend to be recovered in upstream outlets, intermediate loads in mid-stream outlets, and low or near-negative loads in the final outlet.

Cell viability measurements performed after sorting further confirmed that the process remained highly cell-friendly. Using MDA-MB-231 cells as a representative line for viability evaluation, the yield-weighted post-sorting viability was 98.0 ± 1.4% (n = 3). Compared with the pre-chip baseline (~99.8%), the corresponding viability retention *V*_post_/*V*_pre_ was 98.2 ± 1.3% (n = 3) (Table S5), and dead cells were rarely observed in the fluorescence fields of view (Figure 6B). This combination of high recovery, preserved viability, and expression-dependent outlet distributions suggests that the device was suitable as a front-end module for downstream applications—such as culture, imaging, or molecular analysis—that require live, phenotypically stratified cell fractions.

Overall, these results support the central claim of the work: by using soft-magnetic field sculpting and race-based kinematics, a structurally simple, permanent-magnet–biased microfluidic device can perform continuous, expression-level–dependent fractionation of EpCAM-labeled cells with high recovery and viability, while also revealing subtle heterogeneity within each cell line.

## 4. Conclusions and Prospects

In this work, we developed a magnetically sculpted microfluidic platform that performs continuous, expression-level-dependent fractionation of EpCAM-labeled cells. Soft magnetic strips under a uniform permanent-magnet bias are designed using the force-equivalent metric |(***H***·∇)***H*****|**, converting an initially diffuse field into a spatially defined lateral force landscape matched to the microchannel. Combined with a race-based kinematic model and journey-distance analysis, this framework enabled rational placement of three SMS segments and outlet branches to obtain four EpCAM-related magnetic fractions (H, M, L, and N) in a single continuous run.

Simulations and experiments with MCF-7 cells showed that the sculpted field can drive clear race-like lateral deflection and outlet collection, even in a whole-blood/PBS two-phase flow where the interface remained stable. When applied separately to Caco-2, MDA-MB-231, and A549 cells, the device produced distinct outlet distributions consistent with their reported EpCAM expression trends and revealed intra-line heterogeneity, such as an EpCAM-low/negative subset in Caco-2. Across all tests, overall recovery exceeded 90% and post-sorting viability retention (post-/pre-chip) reached 98.2 ± 1.3%, indicating that the magnetic actuation is gentle and compatible with downstream culture or analysis.

The main contribution of this study is methodological: we provide a quantitative field design and kinematic framework that links SMS geometry and bias-field conditions to a predictable lateral force landscape and to outlet positions, rather than proposing a new biological subtype classification. Several aspects remain to be refined, including orthogonal single-cell EpCAM phenotyping (e.g., single-cell immunostaining) and more fully quantitative, protein-normalized assays, and more detailed characterization of the magnetic materials. Nonetheless, the concepts demonstrated here are readily transferable to other markers and cell types that can be magnetically labeled, and can be extended by adapting |(***H***·∇)***H*****|** guided design to new channel geometries, throughput targets, or integrated downstream modules. We anticipate that this approach will be useful as a general front-end strategy for expression-resolved processing in magneto-fluidic systems.

## Data Availability

The original contributions presented in this study are included in the article. Further inquiries can be directed to the corresponding author.

## Supplementary Materials

The following supporting information can be downloaded at: https://www.mdpi.com/article/10.3390/mi17010009/s1, Table S1: Orthogonal experimental design for regulating magnetic field parameters; Table S2: Quantitative definition of outlet groups by Dynabead-load intervals; Table S3: Assessment of RBC/WBC contamination in outlet fractions under whole-blood/PBS two-phase operation; Table S4: Outlet-resolved cell recovery for expression-level fractionation benchmarking; Table S5: Pre- and post-sorting viability assessment of the microfluidic sorting process using MDA-MB-231 cells (n = 3); Figure S1: The magnetic field parameters in the flow channel corresponding to the orthogonal experiment; Figure S2: Sensitivity analyses of kinematic predictions to cell size and start-line confinement; Figure S3: Outlet-resolved distributions of Dynabead loading after continuous subtype fractionation; Figure S4: Phase field distribution in continuous flow sorting chips; Figure S5: Quantitative simulation sweep of total flow rate and its impact on outlet fractionation; Figure S6: Western blot validation of EpCAM expression ranking in outlet-defined MCF-7 fractions; Video S1: Real-time fluorescence video showing race-based lateral deflection and stable co-flow interface in the magnetic control region (corresponding to Figure 5).

## Abbreviations

The following abbreviations are used in this manuscript:

SMS	soft magnetic strip
EpCAM	epithelial cell adhesion molecule
MACS	magnetic-activated cell sorting
SDS-PAGE	sodium dodecyl sulfate–polyacrylamide gel electrophoresis
PVDF	polyvinylidene difluoride
HRP	horseradish peroxidase
