# Supplementary material for: Magnetically Sculpted Microfluidics for Continuous-Flow Fractionation of Cell Populations by EpCAM Expression Level
Source: Micromachines (Basel). 2025 Dec 22;17(1):9. doi: 10.3390/mi17010009 (PMC12844116; doi:10.3390/mi17010009)
Supplement: Supplementary file 1 [file micromachines-17-00009-s001.zip › micromachines-4034659-supplementary.pdf]

Supplementary Materials

Magnetically Sculpted Microfluidics for Continuous-Flow Fractionation of Cell Populations by EpCAM Expression Level

Zhenwei Liang [1], Xiaolei Guo [1,2], Xuanhe Zhang [1], Yiqing Chen [1], Chuan Du [3], Yuan Ma [1,*] and Jiadao Wang [1,*]

[1] Department of Mechanical Engineering, Tsinghua University, Beijing 100084, China
[2] Center for Medical Device Evaluation, National Medical Products Administration, Beijing 100081, China
[3] LenCyte Biotechnology (Xuzhou City) Co., Ltd., Xuzhou 221000, China
* Correspondence: yuanma@tsinghua.edu.cn (Y.M.); jdwang@mail.tsinghua.edu.cn (J.W.)

Table S1. Orthogonal experimental design for regulating magnetic field parameters.

Group	Distance (μm)	Width (μm)	Thickness (μm)
**1**		800	
**2**	50	1000	
**3**		1200	
**4**		800	
**5**	60	1000	100
**6**		1200	
**7**		800	
**8**	70	1000	
**9**		1200	

Table S2. Quantitative definition of outlet groups by Dynabead-load intervals. Microscopy-based bead counting was performed for cells collected from each outlet (H, M, L, and Group N) across three independent runs. Cells were binned into four bead-load intervals ($N \geqslant 8$, $3 \leqslant N \leqslant 7$, $1 \leqslant N \leqslant 2$, and N=0), and the table reports the percentage of cells in each outlet falling into each interval. Under the experimental conditions used here, these intervals provide the most statistically discriminative mapping for labeling levels (H: $N \geqslant 8$; M: 3-7; L: 1 – 2; N: 0).

Group	$N \geq 8$	$7 \geq N \geq 3$	$2 \geq N \geq 1$	N=0
**H-1**	97.5%	2.5%	0.0%	0.0%
**H-2**	98.5%	1.5%	0.0%	0.0%
**H-3**	95.0%	5.0%	0.0%	0.0%
**M-1**	6.0%	92.5%	1.5%	0.0%
**M-2**	12.0%	88.0%	0.0%	0.0%
**M-3**	4.5%	94.0%	1.5%	0.0%
**L-1**	0.0%	10.5%	89.5%	0.0%
**L-2**	0.0%	10.0%	90.0%	0.0%
**L-3**	0.0%	4.5%	95.5%	0.0%
**N-1**	0.0%	0.0%	6.5%	93.5%
**N-2**	0.0%	0.0%	4.0%	96.0%
**N-3**	0.0%	0.0%	5.5%	94.5%

Outlet purity: Outlet purity is defined as the fraction of collected cells that fall within the dominant bead-load interval assigned to that outlet. Based on Table S2 (n = 3 runs), the purities are: H: 97.0 ± 1.8% (95.0–98.5%), M: 91.5 ± 3.1% (88.0–94.0%), L: 91.7 ± 3.3% (89.5–95.5%), and Group N: 94.7 ± 1.3% (93.5–96.0%).

Table S3. Assessment of RBC/WBC contamination in outlet fractions under whole-blood/PBS two-phase operation. Outlet-wise counts of inspected events and detected RBC/WBC (CD45 for WBC; Ter119 for RBC) for H/M/L outlets (n = 3 per outlet). No detectable RBC/WBC contamination was observed. Because the N outlet is intentionally designed to carry the whole-blood phase, we focused the purity assessment on the H/M/L fractions that are used for downstream analysis. Events were counted by fluorescence microscopy of collected fractions after immunostaining (CD45 for WBC; Ter119 for RBC).

Group	Total inspected events	WBC (CD45+)	RBC (Ter119+)	Fraction (%)
**H**	3579	0	0	0
	3682	0	0	0
	4715	0	0	0
**M**	3619	0	0	0
	4006	0	0	0
	3277	0	0	0
**L**	2976	0	0	0
	3151	0	0	0
	3320	0	0	0

Table S4. Outlet-resolved cell recovery for expression-level fractionation benchmarking. Outlet-specific recovery fractions were quantified for Caco-2, MDA-MB-231, and A549 cells (n = 3 runs per line). For each run, the recovery for outlet $k \in \{H, M, L, N\}$ was calculated as $R_k = N_k / N_{in}$, where N_k is the number of cells collected from outlet k and N_{in} is the total number of cells loaded. The "all" column reports the overall recovery $R_{all} = \sum_k R_k$. Values correspond to the same benchmarking experiments summarized in Figure 6A, but are reported here relative to the input cell number to make outlet-specific recovery explicit.

			**Caco-2**		
**run**	H	M	L	N	all
**1**	48.70%	19.50%	14.20%	13.60%	96.00%
**2**	43.30%	27.20%	13.60%	8.70%	92.80%
**3**	39.00%	28.20%	14.00%	6.80%	88.00%
			**MDA-MB-231**		
**run**	H	M	L	N	all
**1**	20.70%	29.00%	28.80%	19.50%	98.00%
**2**	22.70%	28.40%	30.30%	14.90%	96.30%
**3**	21.70%	25.70%	26.10%	20.60%	94.10%
			**A549**		
**run**	H	M	L	N	all
**1**	0.00%	3.60%	16.40%	78.50%	98.50%
**2**	0.00%	0.00%	22.80%	76.30%	99.10%
**3**	0.00%	1.50%	14.00%	80.50%	96.00%

Table S5. Pre- and post-sorting viability assessment of the microfluidic sorting process using MDA-MB-231 cells (n = 3). Baseline viability V_{pre} was measured before chip processing by Calcein-AM/propidium iodide (PI) staining of an aliquot of the prepared suspension. After sorting, outlet fractions (H/M/L/N) were collected and stained under the same conditions to obtain fraction-specific post-chip viabilities $V_{post,i}$. Viability was calculated as the percentage of Calcein-positive / PI-negative cells among all counted cells. An overall post-sorting viability V_{post} was computed as a yield-weighted average of $V_{post,i}$ using the outlet recovery fractions from the corresponding sorting run (a representative outlet distribution is shown in Figure 6A). Viability retention was defined as V_{post}/ V_{pre}. Across three independent runs, V_{pre} was ~99.8%, the yield-weighted V_{post} was 98.0 ± 1.4%, and the resulting viability retention V_{post}/ V_{pre} was 98.2 ± 1.3%, indicating that the sorting process is gentle and compatible with downstream analysis/culture.

**Cell viability Analysis**			
Run	1	2	3
V_{pre}	99.80%	99.65%	100.00%
$V_{post, H}$	97.42%	97.97%	99.63%
$V_{post, M}$	96.31%	97.34%	99.12%
$V_{post, L}$	97.11%	97.92%	99.76%
$V_{post, N}$	95.52%	98.52%	99.73%
yield-weighted V_{post}	96.70%	97.85%	99.54%
V_{post}/ V_{pre}	96.89%	98.19%	99.50%

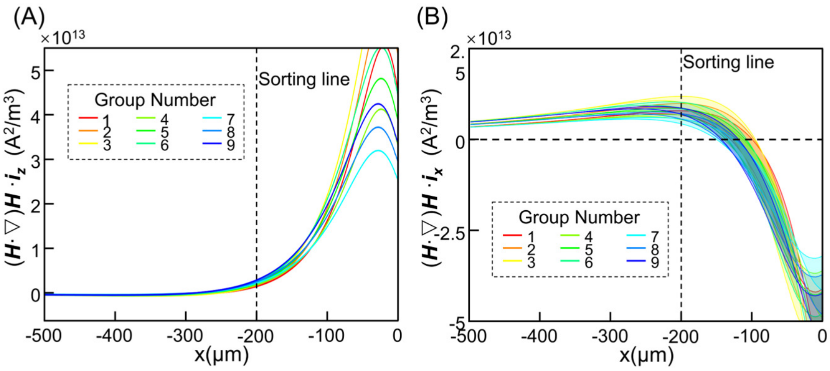

Figure S1. The magnetic field parameters in the flow channel corresponding to the orthogonal experiment. (**A**) The z-axis component of the $(\boldsymbol{H}\cdot\nabla)\boldsymbol{H}$ parameter within the flow channel for the nine design models. (**B**) The x-axis component of the $(\boldsymbol{H}\cdot\nabla)\boldsymbol{H}$ parameter within the flow channel for the nine design models.

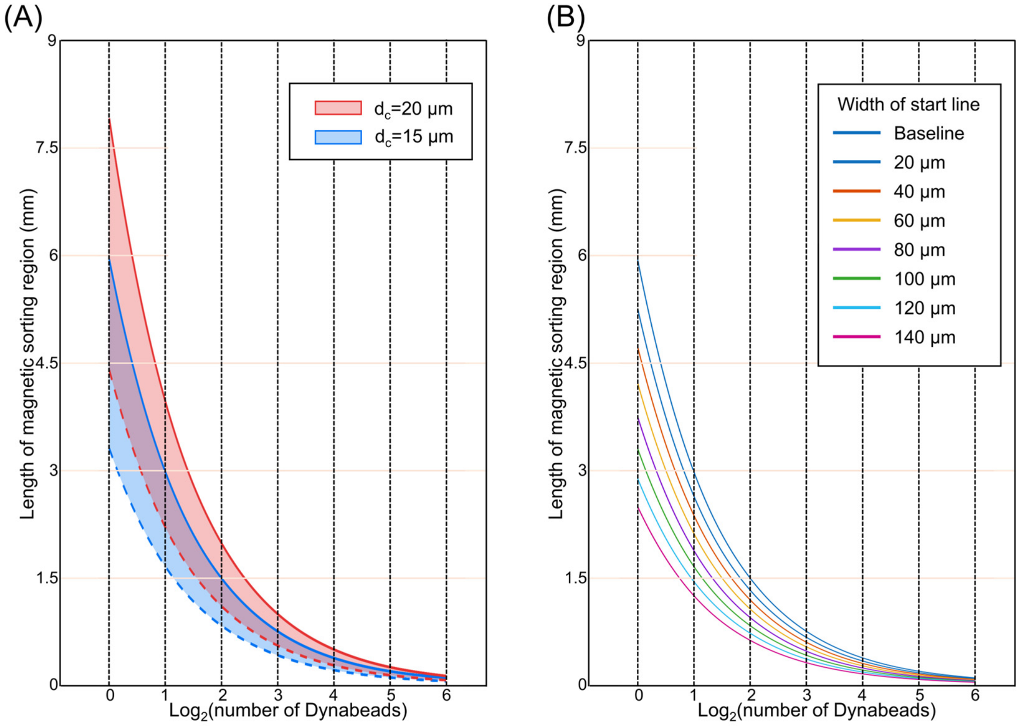

Figure S2. Sensitivity analyses of kinematic predictions to cell size and start-line confinement. (A) Predicted journey-distance bands as a function of bead load ($\log_2(N)$) for two representative cell diameters (d_c = 20 µm and d_c = 15 µm), illustrating that larger cells experience higher hydrodynamic resistance and require a longer magnetic interaction length to reach the same outlet-crossing criterion. The shaded envelopes represent the range of journey distances arising from variations in initial position and near-wall interaction conditions. (B) Systematic sweep of the start-line width (20–140 µm) for d_c = 15 µm cells, used as a proxy for varying the sheath-to-sample ratio. "Baseline" denotes the farthest effective crossing distance within the channel, and the envelope between each curve and the baseline represents the uncertainty range attributable to dispersion in the initial lateral position ("start line"). Narrower start-line confinement yields curves closer to the baseline and a reduced uncertainty envelope, indicating improved robustness in outlet-crossing distances for a given bead load. However, because tighter confinement requires a lower sample fraction under a fixed total flow, the gain in precision comes at the cost of proportionally reduced sample throughput. Therefore, a ~100 µm start line was selected as a practical compromise between sorting precision and usable throughput for experimental benchmarking.

Extended description for panel A (cell-size sensitivity, 15 µm vs 20 µm): Kinematic simulations were repeated under the same magnetic-field landscape, flow conditions, and initialization of virtual cells within the confined sample band as in Figure 4B, with cell diameter d_c varied between 15 µm and 20 µm. Cell size enters the model through the Stokes drag term (Eq. (1)); therefore, at identical Dynabead loading N, a larger d_c increases hydrodynamic resistance and reduces lateral migration speed, leading to longer required interaction/journey distances for crossing the sorting line. Journey distances are plotted versus $\log_2(N)$, and the colored bands represent the spread caused by variations in initial position and near-wall interaction conditions (consistent with the definition in Figure 4B).

Overall, increasing d_c shifts the journey-distance bands upward (i.e., reduced deflection "efficiency") while preserving their monotonic ordering with bead load. Importantly, the bead-load thresholds separating H/M/L shift modestly within the experimentally relevant size range: for d_c = 20 μm cells with $N \geq 5$ predominantly satisfy the H-group criterion, N=3-4 map to M, and N=1-2 map to L; whereas for d_c = 15 μm, the H-group threshold relaxes to $N \geq 4$, with N=2-3 mapping to M and N=1 mapping to L. These results indicate that cell-size variability mainly introduces a small shift in the group-boundary bead thresholds, while the race-based separation mechanism remains governed primarily by magnetic loading.

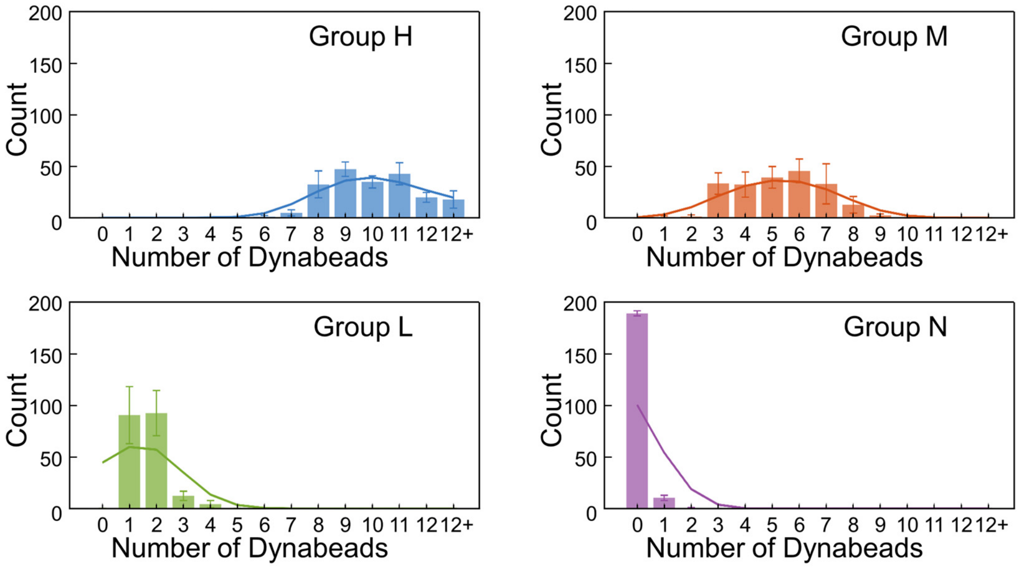

Figure S3. Outlet-resolved distributions of Dynabead loading after continuous subtype fractionation. Dynabead load per cell (N, number of bound Dynabeads per cell; roman "Group N" denotes the near-negative outlet group) was quantified by microscopy after outlet collection. For each outlet fraction, three randomly selected non-overlapping fields of view were analyzed; each field was normalized to 200 cells, and histogram bars are reported as mean counts per bead-number bin with error bars showing mean ± s.d. across the three fields. The distributions exhibit a monotonic shift toward lower bead counts from H → M → L → Group N, confirming that the sorted fractions differ in magnetic loading as intended and supporting the quantitative mapping summarized in Table S2.

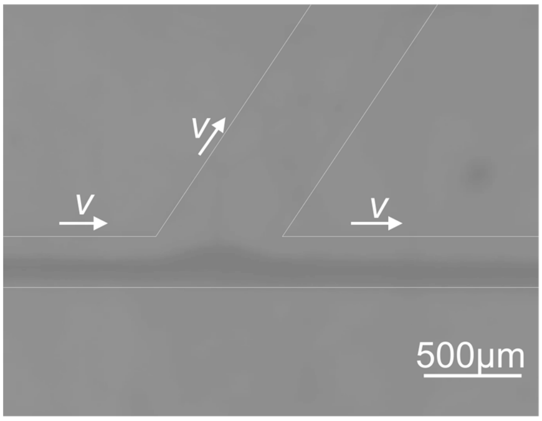

Figure S4. Phase field distribution in continuous flow sorting chips. Whole blood and PBS solution were used as the sample and buffer phases, respectively. The darker region in the figure corresponds to the whole-blood phase. The confined whole-blood phase remains in the main flow path toward the downstream outlet (N), supporting minimal blood-cell entry into the branch outlets.

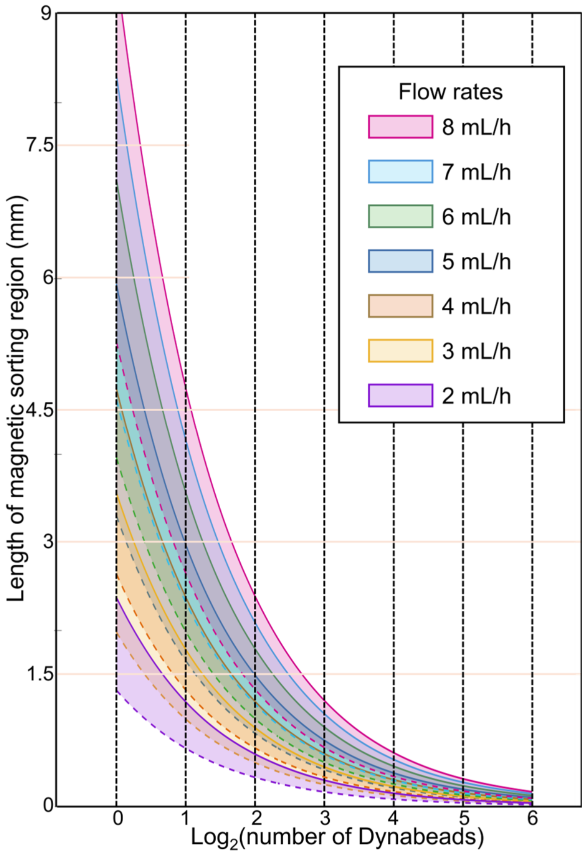

Figure S5. Quantitative simulation sweep of total flow rate and its impact on outlet fractionation. Simulations were performed by varying the total flow rate while keeping the sheath-to-sample flow-rate ratio fixed at 4:1, consistent with the experimental configuration. Increasing the total flow rate reduces the interaction time for lateral magnetic deflection, thereby shifting bead-loading thresholds upward and increasing the required magnetic interaction length. When the total flow rate is increased by 60% (from 5 mL/h to 8 mL/h), boundary leakage for weakly labeled cells begins to appear and the H-capture threshold shifts from $N \approx 5$ to $N \approx 7$. Conversely, decreasing the total flow rate to 2 mL/h shifts thresholds downward and causes over-capture: most bead-labeled cells are predicted to be collected in the H fraction, the L fraction becomes nearly empty, the M fraction mainly collects residual singly labeled cells, and the N fraction continues to collect EpCAM-negative cells. These results define a practical operating window around the nominal 5 mL/h condition used in experiments.

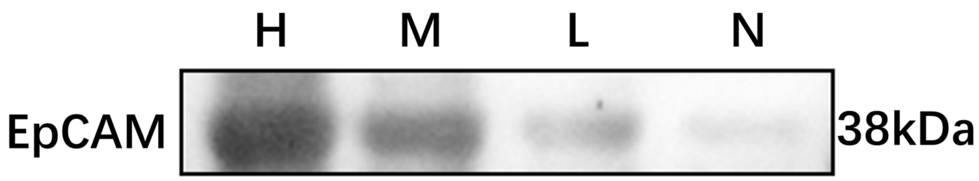

Figure S6. Western blot validation of EpCAM expression ranking in outlet-defined MCF-7 fractions. MCF-7 cells were immunomagnetically labeled with anti-EpCAM Dynabeads and processed through the magnetically sculpted microfluidic sorter, yielding four outlet fractions denoted H, M, L, and N. The Western blot shown here was obtained from a single large-scale sorting run processing 2.0×10^6 total MCF-7 cells, with outlet yields of approximately H $\approx$ 7.5×10^5 (37.5%), M $\approx$ 4.0×10^5 (20.0%), L $\approx$ 6.0×10^5 (30.0%), and N $\approx$ 2.5×10^5 (12.5%). To exclude trivial cell-number effects, an equal number of cells from each fraction (2.5×10^5 cells per lane) was loaded for SDS-PAGE after resuspension and dilution-based sampling from the collected pellets. EpCAM band intensity decreases monotonically from H to N, supporting that the magnetically defined fractions correspond to progressively lower EpCAM signal on a per-cell basis rather than arbitrary outlet assignment. (Because the blot was loaded by equal cell number rather than normalized total protein/housekeeping control, the result is used as supportive, semi-quantitative evidence for the expected trend.)
